# Efficient conformational sampling and weak scoring in docking programs? Strategy of the wisdom of crowds

**DOI:** 10.1186/s13321-017-0227-x

**Published:** 2017-06-12

**Authors:** Ludovic Chaput, Liliane Mouawad

**Affiliations:** 1 Chemistry, Modelling and Imaging for Biology (CMIB), Institut Curie - PSL Research University, Bât 112, Centre Universitaire, 91405 Orsay Cedex, France; 2 Paris-Sud University, Orsay, France; 3 Inserm, U1196, Orsay, France; 4 CNRS, UMR 9187, Orsay, France; 5 Selebio SAS, 17 rue de la Barauderie, 77140 Darvault, France

**Keywords:** Docking, Rescoring, USC, Gold, Glide, Surflex, FlexX, DUD-E, Benchmark

## Abstract

**Background:**

In drug design, an efficient structure-based optimization of a ligand needs the precise knowledge of the protein–ligand interactions. In the absence of experimental information, docking programs are necessary for ligand positioning, and the choice of a reliable program is essential for the success of such an optimization. The performances of four popular docking programs, Gold, Glide, Surflex and FlexX, were investigated using 100 crystal structures of complexes taken from the Directory of Useful Decoys-Enhanced database.

**Results:**

The ligand conformational sampling was rather efficient, with a correct pose found for a maximum of 84 complexes, obtained by Surflex. However, the ranking of the correct poses was not as efficient, with a maximum of 68 top-rank or 75 top-4 rank correct poses given by *Glidescore*. No relationship was found between either the sampling or the scoring performance of the four programs and the properties of either the targets or the small molecules, except for the number of ligand rotatable bonds. As well, no exploitable relationship was found between each program performance in docking and in virtual screening; a wrong top-rank pose may obtain a good score that allows it to be ranked among the most active compounds and vice versa. Also, to improve the results of docking, the strengths of the programs were combined either by using a rescoring procedure or the United Subset Consensus (USC). Oddly, positioning with Surflex and rescoring with *Glidescore* did not improve the results. However, USC based on docking allowed us to obtain a correct pose in the top-4 rank for 87 complexes. Finally, nine complexes were scrutinized, because a correct pose was found by at least one program but poorly ranked by all four programs. Contrarily to what was expected, except for one case, this was not due to weaknesses of the scoring functions.

**Conclusions:**

We conclude that the scoring functions should be improved to detect the correct poses, but sometimes their failure may be due to other varied considerations. To increase the chances of success, we recommend to use several programs and combine their results.

**Graphical abstract:**
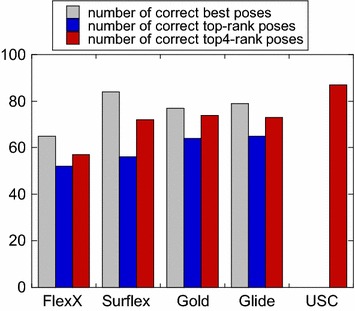
Summary of the results obtained by semi-rigid docking of crystallographic ligands. The docking was done on 100 protein-ligand X-ray structures, taken from the DUD-E database, and using four programs, Glide, Gold, Surflex and FlexX. Based on the docking results, we applied our United Subset Consensus method (USC), for which only the top4-rank poses are relevant. The number of complexes for which the best pose is correct, is represented by the gray boxes, the blue and red boxes correspond to the number of complexes with a correct pose ranked as the top 1 or within the top 4. A pose is considered correct when its root-mean-square deviation from the crystal structure is less than 2 Å

**Electronic supplementary material:**

The online version of this article (doi:10.1186/s13321-017-0227-x) contains supplementary material, which is available to authorized users.

## Background

In drug discovery campaigns, hit-to-lead is the stage during which the binding affinity between a newly identified molecule and a therapeutic protein target is optimized. A structure-based optimization necessitates the precise knowledge of the interactions between the ligand and the protein in order to improve the ligand affinity in an efficient and rational way. This knowledge is best obtained by resolving the structure of the protein–ligand complex, either by X-ray crystallography or by NMR, which is time consuming and sometimes difficult to achieve, especially if the binding mode of several compounds has to be investigated. Hence the use of docking programs, which could be precious tools for the identification of the binding modes considering their ease of use and rapidity. However, despite the continuous improvements brought to these programs, the predicted binding modes of the ligands are still far from being absolutely reliable [1].

Both commercial and non-commercial docking programs are available, Autodock [2], Gold [3], Glide [4], Surflex [5] and FlexX [6] being the most used [1]. While Autodock, an open-source program, is popular in academic research groups, private companies usually prefer commercial solutions such as Gold, Glide, Surflex and FlexX, for their speed and efficiency. Here, we only consider the last four programs. Benchmarks of docking programs are regularly released to compare their performance, whether by using semi-rigid docking (flexible ligand, rigid protein) [7–14], flexible docking (flexible ligand and protein) [15–17], ensemble docking (using several conformations of the protein) [15, 18] or cross-docking (docking ligands taken from some structures on proteins from other structures) [19, 20]. We may cite more particularly some semi-rigid docking benchmarks that include Gold, Glide, Surflex or FlexX and that were made on a large number of targets. Usually, benchmarks are used to compare, or simply evaluate, the performance of programs or scoring functions. For example, Liebeschuetz et al. [21] evaluated the pose prediction and the performance of Gold scoring functions using the Directory of Useful Decoys (DUD) dataset [22], which consists of 40 varied protein targets. They found that 81% of the top-rank poses were close to the crystal conformation with a root-mean-square deviation (RMSD) lower than 2 Å. Recently, Murphy et al. [23] investigated, by an ensemble docking, the performance of various scoring functions in Glide for docking and virtual screening on 22 targets and obtained 92% of the top-rank poses within 2 Å from the crystal pose. In other cases, benchmarks are also used to correlate the program performance to the protein or ligand properties. For instance, Kellenberger et al. [24] evaluated the ability of six docking programs to recover the X-ray pose for 100 protein–ligand complexes. They reported that Gold, Glide, Surflex and FlexX were the most accurate programs and that generally their performance decreased with the size of the binding site, the size of the ligand and the number of its rotatable bonds. Some of these observations were corroborated by Li et al. [12] on 195 protein–ligand complexes. On the other hand, Wang et al. [25] have reported from a comprehensive evaluation of ten docking programs that the correlation between the scores of some programs (Gold, Glide and Surflex, among others) and the binding affinities may be high for certain protein families (up to 0.7). They concluded that these programs may be more suitable for these families. However, this assertion seems fragile in the absence of information concerning the degree of identity between the proteins of the same family and the similarities between their ligands.

Some conclusions of these docking benchmarks seem to be in contradiction with our observations made on a virtual screening benchmark [26]. Indeed, we have reported recently the results of the benchmark of Gold, Glide, Surflex and FlexX for virtual screening on the 102 protein targets of the Directory of Useful Decoys-Enhanced database (DUD-E) [27]. We have shown that the good performance of these programs was mainly due to biases in some properties of the chemical libraries and that in contrast, there was no clear relationship between the performance and the properties of the protein cavities or the protein families. The reasons for these discrepancies should be investigated. Are they solely due to the difference between docking (i.e., the comparison between poses of the same molecule) and virtual screening (i.e., the comparison between the top-rank pose of several different molecules)? Since the first is the basis of the second, we may ask if there is a relationship between the performances of these programs in docking and in virtual screening and what would be the influence of the protein and ligand properties on the results of docking. Finally, the weaknesses of the scoring functions were described to be responsible for the docking bad performance of some programs. Is this always the case? And is there a strategy to overcome this bad performance?

To answer these questions, we performed docking calculations on the crystal structures of 100 protein–ligand complexes taken from the DUD-E dataset, using Gold, Glide, Surflex and FlexX. Since only the semi-rigid docking is common to these programs, it was adopted here for the sake of comparability. In what follows, the docking performance of the four programs is first evaluated; then, for each program, the relationship between the performance and the properties of the proteins and the small molecules is investigated and the docking and virtual screening performances are compared; finally, two procedures to improve the chances of obtaining correct poses are explored. But beforehand, the pertinence of the protein–ligand database is investigated to assess its representativeness.

## Results and discussion

### Pertinence of the database for docking

The DUD-E database is made of 102 diversified targets. Two of these proteins, aofb and casp3, are covalently bound to their crystal ligand, so they were excluded from the dataset because they cannot be properly handled by the docking programs in their standard usage. The 100 remaining target structures were resolved by X-ray crystallography, at a resolution ranging between 0.97 and 3.3 Å (Additional file 1: Fig. S1), with a majority around 2 Å, indicating that the crystal poses have a good chance to be well defined, with an uncertainty on their position lower than 1 Å [28]. Of these proteins, only 58 could be gathered in 9 varied families [26]: protein kinases, nuclear receptors, proteases, GPCRs, cleaving enzymes, cyclooxygenases, cytochromes P450, ion channels and histone deacetylases. The others were unique samples gathered in a miscellaneous set. In addition, their binding sites were shown to be diverse considering their size (i.e., the total number of heavy atoms in the cavity), their hydrophobicity (represented by the fraction of carbon atoms, FCA, in the cavity) and their exposure to the solvent (Additional file 1: Fig. S1). Regarding the crystal ligands, some of their physicochemical properties were calculated: the molecular weight (MW), the octanol/water partition coefficient (AlogP), the polar surface area (PSA), the embranchment count (EC), the number of hydrogen bond acceptors (HBA), the number of hydrogen bond donors (HBD), the ring count (RC) and the number of rotatable bonds (RB). For more details, see “Methods” section and Ref. [26]. The distribution of these properties, spreading on a wide range (Additional file 1: Fig. S2), shows a good diversity of the ligands. All these considerations make this dataset of 100 protein/ligand complexes adequate for the assessment of the docking programs.

### Evaluation of the docking programs

All docking programs follow the same general scheme consisting of two main steps: first, the program generates a large set of poses by exploring multiple conformations of the ligand into the binding site, with a rough evaluation to reject the most unrealistic ones, and second, the retained poses are more finely evaluated and ranked using a scoring function. The four programs considered here have different search algorithms. Glide [4, 29] relies on a rough systematic search in a cuboid grid, followed by a refinement using Monte Carlo sampling. The pose generation of Surflex [5] and FlexX [6] is based on an incremental construction algorithm where the small molecule is decomposed in fragments that the program attempts to place in the binding site. The particularity of Surflex is that the positioning of the fragments uses a protomol that fits the site surface. The conformational search method of Gold is based on a genetic algorithm. Considering the scoring functions, *Surflex* is mostly based on empirical energetic terms [30]. *Glidescore*, *FlexX* and *Piecewise Linear Potential* (*PLP*, one of the Gold scoring functions) derive from the empirical *ChemScore* function [31]. *Goldscore* (another Gold scoring function) [3, 32] is a sum of empirical terms and force field-like terms for van der Waals and Coulombic energies. In Glide, *Emodel* [4] combines *Glidescore* and nonbonded energy terms.

For each program, 30 ligand poses per protein target were requested. With Glide the two available conformational sampling methods were used: the standard precision (SP) and the extra-precision (XP) methods. The obtained poses were ranked using several scoring functions, when available, i.e., either *Glidescore* or *Emodel* for Glide and either *PLP* or *Goldscore* for Gold. Our analyses focus on both the ability of the programs to generate a correct docking pose and to top-rank it. To assess the quality of the poses, the root-mean-square deviation between them and the conformation of the corresponding ligand in the crystal structure was used, based on heavy atoms. This RMSD calculation takes into consideration the symmetry of the ligands (see “Methods” section for more details). A pose is considered correct or good when its RMSD from the crystal structure is less than 2 Å, a value corresponding to thermal fluctuations. The summary of the results, given in Table 1, shows that, although a total of 3000 poses were requested for the 100 targets, none of the programs did generate and retain such a number. The obtained poses ranged between 392, for Glide-XP, and 2915, for FlexX. However, a high number of poses does not guarantee their good quality. For instance, there are a little more poses generated with FlexX than with Surflex (2915 and 2899, respectively), but significantly fewer correct poses (910 against 1152) and these poses belong to less targets (65 and 84, respectively). Additionally, the generation of good poses does not guarantee their good ranking. For instance, Surflex generated much more correct poses than Glide-XP (1152 against 210), belonging to significantly more targets (84 vs 74). However, the number of targets for which a good pose is ranked in the top 4 is slightly smaller for Surflex than for Glide-XP (72 vs 73) and the gap is widened for the top 1 pose (56 against 68).

**Table 1:** Comparison of the efficacy of the programs

Programs: sampling methods and *scoring functions*	Conformational sampling			*Scoring*	
	1-Number of poses obtained	2-Number of correct poses	3-Number of targets with a correct pose	*4*-*Number of targets with a correct pose ranked in the top 4*	*5*-*Number of targets with a correct pose ranked as the top 1*
FlexX	2915	910	65		
*FlexX*				57	52
Surflex	2899	1152	84		
*Surflex*				72	56
Glide-SP	2393	624	79		
*Glidescore*				75	65
*Emodel*				72	65
Glide-XP	392	210	74		
*Glidescore*				73	68
*Emodel*				73	66
Gold	1447	330	77		
*PLP*				74	64
*Goldscore*				73	60
USC based on docking results				87	

The comparison is made for ligand conformational sampling (columns 1–3) and pose scoring (columns 4 and 5). From the requested 3000 poses per program (30 poses per target, for 100 targets), the number of obtained poses is given in the first column. Of these poses a certain number is correct, with RMSD < 2 Å from the crystal position, (column 2) and corresponding to a number of targets (column 3). The number of targets whose correct poses are ranked in the top 4 are given in column 4 and those whose correct poses are top-ranked are given in column 5. The number of targets with a correct pose obtained with the USC method, based on the docking results, is reported in the last line (see the “USC method” section below)

These preliminary results suggest that the efficacy of the programs for the sampling procedure may be ordered as follows: Surflex > Glide-SP > Gold > Glide-XP > FlexX, and for the top-ranking, the scoring functions efficacy as follows: *Glidescore* ≥ *Emodel* > *PLP* > *Goldscore* > *Surflex* > *FlexX*.

A more detailed comparison between the scoring functions of the same program, i.e., *Glidescore* and *Emodel* on the one hand and *PLP* and *Goldscore* on the other hand, is given in Additional file 1: Fig. S3, upper panels. It shows that *Glidescore* > *Emodel* and confirms that *PLP* > *Goldscore.* Therefore, in the rest of the work, unless otherwise stated, for Glide, Glide-SP with *Glidescore* will be considered, and for Gold, the scoring function *PLP* will be taken into account.

**Fig. 1:**
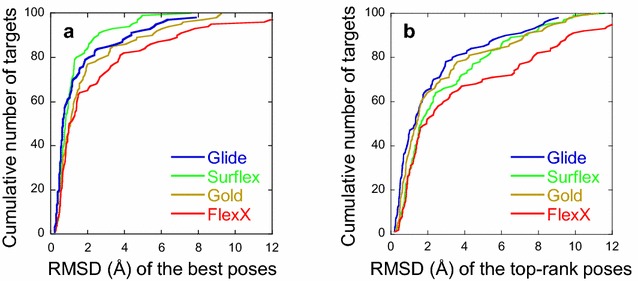
Evaluation of the performance of the programs. The cumulative number of targets with respect to the RMSD of the best poses (**a**) and to the top-rank poses (**b**) obtained by each program

**Fig. 2:**
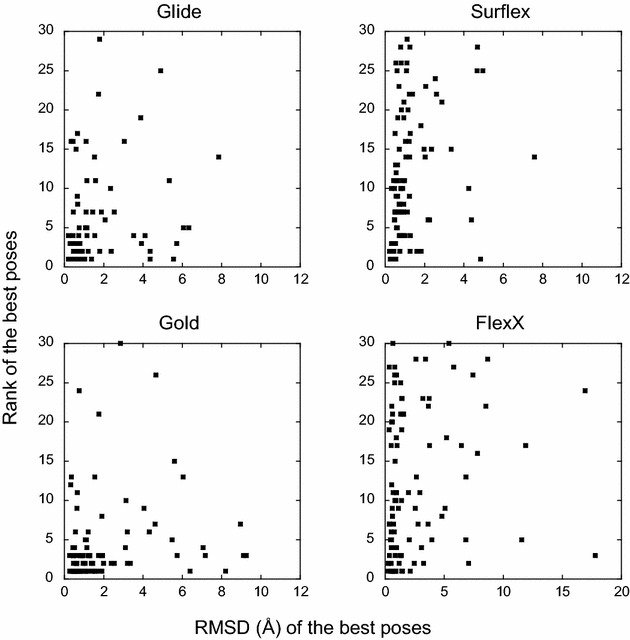
Evaluation of the ability of the programs in detecting the correct poses. For each target, the rank of the best pose is reported with respect to its RMSD from the crystal structure. The rank can range between 1 and 30, since there are at most 30 poses/target. For Glide, Surflex and Gold the RMSDs are less than 10 Å, whereas for FlexX they can reach 18 Å

More detailed analyses are presented in Fig. 1, where the cumulative number of targets with respect to the RMSD of either the best poses (with the smallest RMSD) or the top-rank ones (rank 1) are shown. They confirm the order of efficiency of the programs in finding correct poses and the degradation of the results when identifying them, i.e., when the top-rank poses are considered. Note that for Glide and Surflex, for all targets, the best pose has an RMSD under 8 Å and for Gold under 10 Å. For these three programs, the best poses with the highest RMSDs correspond to ligands that occupy correctly the cavity as to their shape, but with top-to-tail positions relative to the crystallographic ones. By contrast, for FlexX, the best poses may reach an RMSD of 18 Å, corresponding to positions outside of the binding site, at the edge of the cavity. However, in all cases, even when a program succeeds in finding good poses, their ranking may still be unsatisfactory. Indeed, the rank of the best poses ranged between 1 and 30 with all programs. As observed in Fig. 2, being a correct best pose, with RMSD < 2 Å, does not prevent from being in the bottom of the ranking. This is especially true for Surflex and FlexX, where 48 and 46% of the correct best poses, respectively, are placed beyond the 10^th^ rank. For Glide and Gold, these numbers fall to 13 and 8%, respectively.

**Fig. 3:**
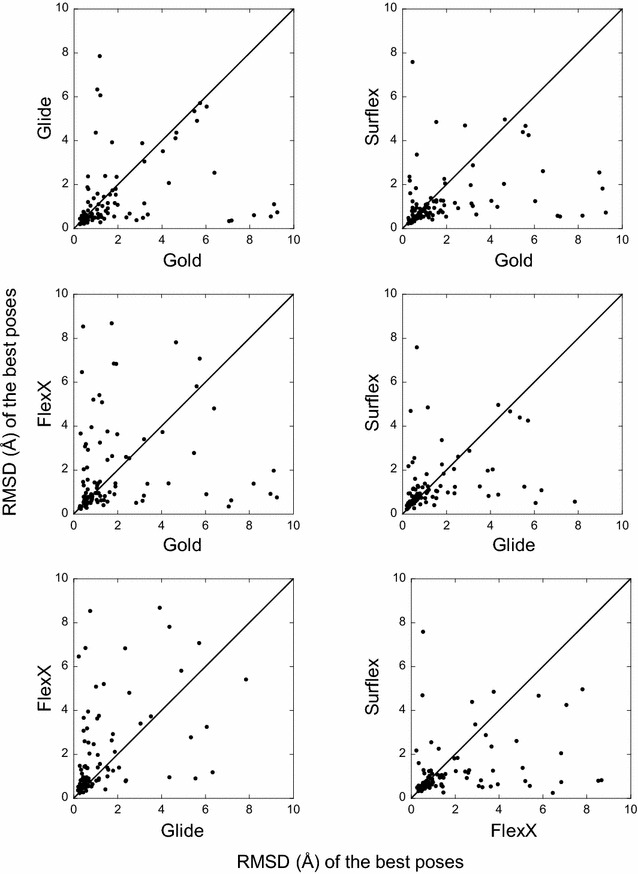
Comparison between the best poses obtained by the four programs. In *each panel*, for each target is reported the RMSD of the best pose obtained by a program with respect to the RMSD of the best pose obtained with another program. If the results were correlated, the points would follow the *diagonal line*. When the points are more dispersed under the diagonal, the program corresponding to the X-axis presents a weaker performance as to finding a correct pose than the program of the Y-axis, and vice versa

**Fig. 4:**
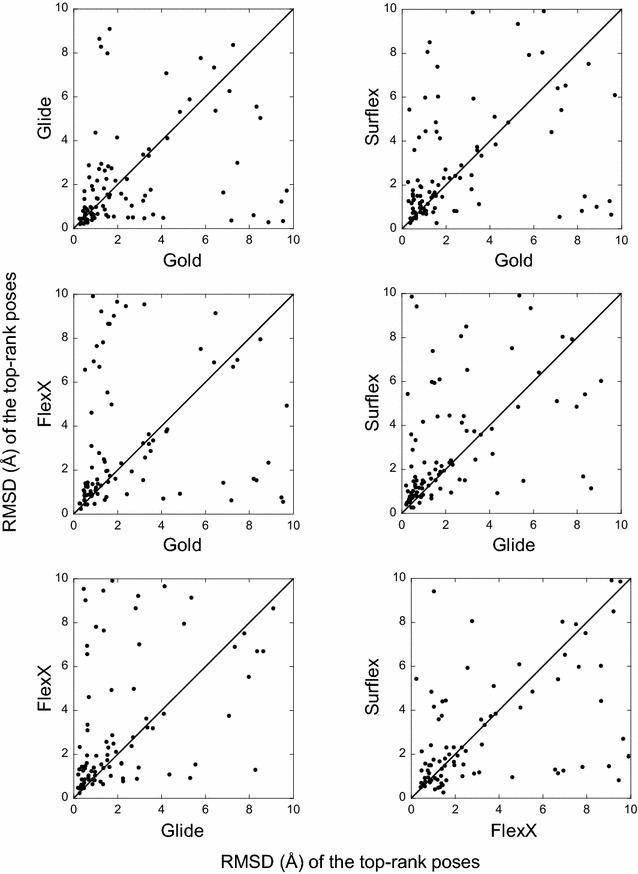
Comparison between the top-rank poses obtained by the four programs. Similar to Fig. 3 but for the top-rank poses

For each target, the best poses (or the top-rank ones) obtained with one program were compared to those obtained with another program. This comparison shows the absence of correlation between the results (Figs. 3, 4). Indeed, for many targets, while a program finds a best pose with an RMSD lower than 2 Å (or top-rank it), another program would be completely unable to find any correct pose for the same target. For instance, if we consider the two programs with comparable performances, namely Glide and Gold, whose best poses were correct for 79 and 77 targets, respectively, they have in common only 67 targets with correct poses, not 77 as could be expected.

Despite the lack of correlations, for 28 targets, all programs succeeded in finding a correct pose and to top-rank it. So these targets will be referred to as “easy”. On the contrary, there were 6 “hard” targets, for which the search algorithms of all programs failed in finding any correct pose. The list of all targets is given in Additional file 2: Table S1, where the easy target cells are colored in green and those of the hard targets in red.

### Is the success of docking influenced by some protein or ligand properties?

In order to identify the exogenous factors that could influence the pose prediction accuracy, the protein and ligand properties presented above were used, i.e., for the targets, two properties concerning the protein in its entirety (the resolution of the crystal structure and the protein family), and three properties concerning solely the binding site (the size of the cavity, its FCA and its exposure to solvent), in addition to eight properties of the ligands (MW, ALogP, PSA, EC, HBA, HBD, RC and RB).

The easy targets have nothing in common, neither the properties of the target itself nor those of the corresponding ligand. Indeed, there is no significant difference between these properties and those of all other targets, or more importantly, of those of the hard targets, as given by either the Student *t*-test or the Mann–Whitney–Wilcoxon test, according to the normality of the property distribution, with a significance threshold of 1% (see Additional file 1: Fig. S4). All the properties are reported in Additional file 2: Table S1.

**Fig. 5:**
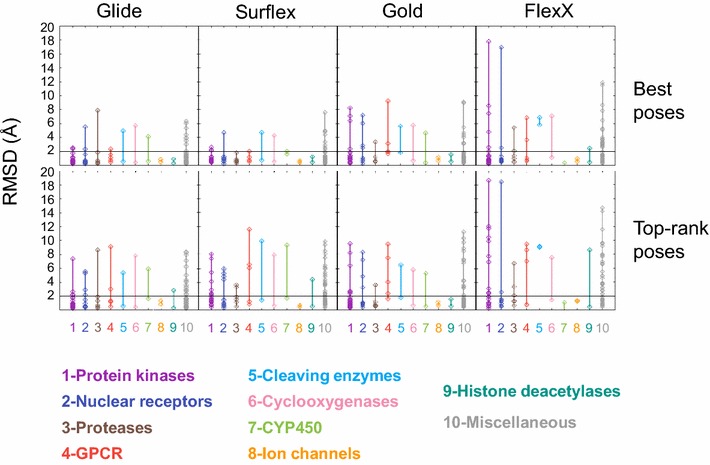
Relationship between the docking results and the protein families. The RMSD of the best poses, *Upper panels*, and the top-rank poses, *Lower panels*, with respect to the protein families. The *color code* of the families is given in the figure. The *horizontal lines* at RMSD = 2 Å delimit the correct poses

There is no obvious relationship between the protein families and the success of the programs in finding the correct pose or in top-ranking it (Fig. 5). Indeed, concerning the top-rank poses obtained with any of the four programs, in a given family there are members with high and others with low RMSD, apart from a few exceptions, like the ion channels (2 targets) for which all programs succeeded (low RMSD). A similar observation can be made concerning the best poses: for most families, the programs succeeded for some targets but not for others, like the nuclear receptors (11 targets), with Surflex, and the proteases (6 targets), with Glide. This is true, despite the existence of some families for which a given program succeeded for all their members, like the proteases (6 targets) with Surflex.

For all the other protein or ligand properties, the Spearman correlation coefficients between the descriptors and the RMSD of both the best poses and the top-rank ones were calculated (Table 2). This correlation was adopted because the descriptors do not follow normal distributions and we do not presume the existence of linear relationships between them and the RMSDs. The correlation is considered as significant when the *p*-value is less than 10^−3^, the limit over which, in our case, a relationship between the variables is not visible in the plots.

**Table 2:** Spearman correlation between the programs performance and the properties of the proteins or the ligands

	Glide	Surflex	Gold	FlexX
A-best pose				
Properties of the proteins				
Crystal structure resolution	NSC	NSC	NSC	NSC
Cavity size^a^	NSC	NSC	NSC	NSC
Hydrophobicity^b^	NSC	NSC	NSC	NSC
Exposure	NSC	NSC	NSC	NSC
Properties of the ligands				
MW	0.34 (6 × 10^−4^)	NSC	0.33 (8 × 10^−4^)	NSC
AlogP	NSC	NSC	NSC	NSC
PSA	NSC	NSC	NSC	NSC
EC	NSC	NSC	NSC	NSC
HBA	NSC	NSC	NSC	NSC
HBD	NSC	NSC	NSC	NSC
RC	NSC	NSC	NSC	NSC
RB	0.47 (<10^−4^)	0.42 (<10^−4^)	0.50 (<10^−4^)	NSC
B-top-rank				
Properties of the proteins				
Crystal structure resolution	NSC	NSC	NSC	NSC
Cavity size^a^	NSC	NSC	NSC	NSC
Hydrophobicity^b^	NSC	NSC	NSC	NSC
Exposure	NSC	NSC	NSC	NSC
Properties of the ligands				
MW	0.34 (7 × 10^−4^)	NSC	NSC	NSC
AlogP	NSC	NSC	NSC	NSC
PSA	NSC	NSC	NSC	NSC
EC	0.34 (7 × 10^−4^)	NSC	NSC	NSC
HBA	NSC	NSC	NSC	NSC
HBD	NSC	NSC	NSC	NSC
RC	NSC	NSC	NSC	NSC
RB	0.46 (<10^−4^)	0.35 (3 × 10^−4^)	0.48 (<10^−4^)	NSC

The correlations are calculated between the properties of the targets or the ligands and the RMSD of the best poses (A) obtained by docking with the 4 programs or the top-rank poses (B). The *p*-values are in parentheses. The correlations are considered non-significant for *p*-value ≥ 10^−3^, and therefore they are omitted

*NSC* no significant correlation

^a^Cavity size = total number of the cavity heavy atoms at 4 Å from the surface

^b^Hydrophobicity of the cavity = fraction of carbon atoms (FCA) among the cavity heavy atoms

We observe that none of the programs performance are correlated to the protein properties. The performance of FlexX is not even correlated with any property of the ligands. Glide, Surflex and Gold results are impacted by the number of rotatable bonds, for either the scoring or the conformational sampling of the small molecules. Indeed, the performance is degraded (increasing RMSD) with increasing number of RBs. In addition, there are also small correlations of MW with Glide and Gold performances, and the scoring function of Glide with the number of embranchments (EC) of the small molecules. The correlation with MW may be due to the increasing number of RBs for larger molecules, which is reflected by the significant correlation between MW and RB (0.63). Therefore, except for the number of rotatable bonds that may make the sampling and scoring of the ligand difficult with Glide, Surflex and Gold, there is no real influence of the properties of the protein or the ligand on the success of the four programs in finding the right pose.

The influence of some of the properties considered here on the performance of the four docking programs was reported in the literature. We will cite the results obtained on Gold and Glide by Perola et al. [8], Kellenberger et al. [24] and Li et al. [12], on Surflex by Li et al. [12] and Kellenberger et al. [24] and FlexX by only Kellenberger et al. [24]. In these works, for Gold, Glide, Surflex and FlexX, the docking accuracy seemed to increase for ligands with higher percentage of buried solvent accessible surface area (SASA) and to decrease with the number of rotatable bonds. Li et al. [12] also investigated the effect of the binding pocket hydrophobicity and reported that the performance of Glide and Surflex is better for indisputably hydrophobic or hydrophilic binding pockets than for pockets with intermediate hydrophobicity, while Gold tends to perform better on only hydrophobic pockets. Kellenberger et al. [24] reported that Gold, Surflex, and FlexX perform better on small binding sites (<700 Å^3^), while Glide performs better on medium binding sites (700–1500 Å^3^), and that all programs have relatively better performance for small hydrophobic ligands. Except for the rotatable bonds, these results may seem in contradiction with our conclusions about the influence of the protein and ligand properties. These apparent discrepancies are merely due to the difference between our approach, which consists of calculating the correlation between the properties and RMSDs, and the approach presented in these articles, which consists of clustering the properties into discrete groups and considering the percentage of good RMSDs in each group. If we had adopted the latter approach, our results would have been in complete agreement with the published ones, but we preferred not to do so, because the groups are not equally populated, neither in our case nor in the cited articles. Indeed, the groups at the edges are much less representative, which may yield misleading conclusions, especially in the absence of statistical significance estimations of the differences among these groups. Note that one of the indicators used in the cited articles, namely the percentage of the ligand SASA, is not explicitly present here. However, this indicator is comparable to the binding site exposure, which represents the openness of the cavity, since the more the cavity is closed, the more the ligand is buried. We preferred the exposure of the binding site to the percentage SASA of the ligand, because the latter is based on the prior knowledge of the structure of the protein–ligand complex, which is far from being the case when docking is needed.

### Comparison with virtual screening

**Fig. 6:**
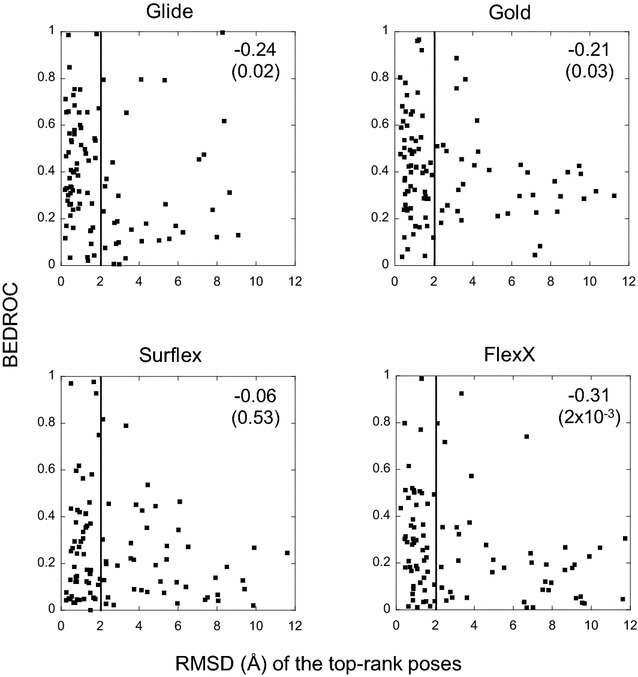
Comparison between the top-rank poses and the virtual screening results. For each target, the BEDROC score, which represents the performance of the virtual screening, is given with respect to the RMSD of the top-rank pose. No comparison was made with the best pose because only the top-rank pose is used for the VS ranking of the molecules. The *vertical lines* at 2 Å delimit the correct poses. For the sake of comparability, all the top-rank poses of FlexX that were beyond 12 Å were omitted. The Spearman correlations with the *p*-values in parentheses are given in the *right upper corner* of *each panel*

The poor correlations observed above contrast with the observations based on the results of the virtual screening (VS) that we performed recently with these four programs, using the same procedure (except for Gold, where the scoring function *Goldscore* was used in the virtual screening study) [26]. In VS, it was observed that the performances (BEDROC scores) of all four programs are somehow influenced by the small molecule properties. Therefore, one may ask if there is any relationship between the results of the docking presented here and those of the virtual screening. To answer this question, for each target, the BEDROC score obtained with a program is plotted *versus* the RMSD of its top-rank pose (Fig. 6), because only this pose is taken into account in VS ranking and therefore in the BEDROC score. This comparison should be taken with its limitations, because in docking we only consider one ligand whereas in VS we consider an entire chemical library. However, this comparison is still legitimate, because docking is the basis of VS and in our case, all VSs were done on the target crystal structures that were used here for docking. Concerning Gold, the RMSD of the poses ranked with *Goldscore* is used in this subsection since only this scoring function, which yielded better results than *PLP* for VS, was adopted for the BEDROC scores. In Fig. 6, it can be observed that, whatever the program, there is no exploitable correlation between the results of docking and VS. For FlexX this correlation is higher than the others, but it mainly concerns weak results in both methodologies. If we only consider the targets with successful VS (BEDROC > 0.5), Glide and Gold failed in docking (RMSD ≥ 2) for 21 and 23% of them, respectively. This number of targets raises to 27% for Surflex and 36% for FlexX, but in this case the total number of targets with successful VS is much smaller than for Glide and Gold. So for the four programs, the relationship between docking and VS is not exploitable in practice. Indeed, an excellent VS result may be obtained with poor docking poses, and vice versa. This can be illustrated by the case of the target pur2, which obtained with Glide an excellent BEDROC score close to 1.0, the best possible score. In this VS, the ligand that was used in the crystal structure was ranked among the most active molecules, despite its top-to-tail pose with respect to the X-ray position, with an RMSD of 8.3 Å. On the opposite, there are numerous targets with excellent docking results, whose RMSD of the top-rank pose is close to zero, which obtained a very poor BEDROC score, close to zero, the worst score. This shows that, contrary to a widespread belief, improving docking does not necessarily help in improving VS, at least with the semi-rigid procedure (flexible ligand and rigid protein).

These observations are in good agreement with those of Cummings et al. [9], who reported the results of virtual screening on 5 different targets and docking on 31 protein–ligand complexes corresponding to these targets. The ligands in the complexes were included in the VS chemical libraries. In this case too, no correlation was observed between the rank of the compounds in VS and the RMSD of ligands with respect to the X-ray poses. However, all these observations do not exclude that, for some cases, it may happen that the performance of VS is improved when good poses are found by docking [33].

### Improving the chance of identifying the correct pose

As observed above, there are programs with high efficiency in finding correct poses, like Surflex, which correctly positioned the ligand for 84 targets, but only identified (or top-ranked) 56 among them, whereas other programs are maybe less efficient in finding correct poses, but with better scoring functions, like *Glidescore*, which was able to top-rank correct poses for 65 targets over the 79 that were well-positioned (Table 1). Therefore, the combination of the skills of these two programs, or others, may improve the docking results. Two different ways of combining the results were tested, the standard rescoring procedure and the United Subset Consensus (USC) described in [34].

#### Rescoring

The poses obtained by each program were rescored using all the scoring functions considered in Table 1. The soft rescoring procedure, which slightly optimizes the ligand to accommodate the pose that was provided from another program, was applied (see Methods for details). For the sake of homogeneity and comparability between the programs, the poses obtained by docking with a program were also rescored with the same program. Rescoring with *Emodel* did not produce any improvements to the ranking and was therefore discarded again. However, rescoring with *Goldscore* gave clearly better results than with *PLP*, as observed in Additional file 1: Fig. S3 lower panels, therefore, *Goldscore* was adopted for rescoring. The results of the retained functions (*Glidescore*, *Surflex*, *Goldscore* and *FlexX*) are given in Table 3A and B.

**Table 3:** Assessment of the rescoring results

Sampling	Rescoring			
	*Glidescore*	*Surflex*	*Goldscore*	*FlexX*
A—Number of correct top-rank poses				
Glide	65	58	70	63
Surflex	63	56	64	67
Gold	64	63	62	62
FlexX	51	58	56	50
Pool	75	63	73	64

Sampling	Rescoring				
	*Glidescore*	*Surflex*	*Goldscore*	*FlexX*	USC
B—Number of correct top-4 rank poses					
Glide	76	74	75	71	75
Surflex	73	72	75	75	74
Gold	71	70	71	69	70
FlexX	62	63	62	57	63
Pool	82	79	86	72	85

Number of targets whose top-rank poses (A) or top-4 rank poses (B) are correct after rescoring. Either all poses obtained with a given program were rescored with all programs (lines 1–4) or all poses were grouped in one pool and rescored with all programs (last line). The results of USC are reported in the last column of (B)

After rescoring, only small improvements in the results were observed comparatively to the docking. Indeed, whereas after docking the number of targets with a correct top-rank pose (RMSD < 2 Å), obtained by the retained sampling/scoring methods, was in the interval of values [52, 65] for the four programs (Table 1, column 5), after rescoring, this number ranged in the interval [50, 70] as observed in Table 3A, lines 1–4. In addition, contrarily to what was expected, conformational sampling with Surflex and rescoring with *Glidescore* yielded rather poor results, with correct poses top-ranked for only 63 targets. Note that the best result, with 70 correct top-rank poses, was obtained using the sampling with Glide and the rescoring with *Goldscore*.

For the top-4 poses (Table 3B), i.e., when at least one correct pose is ranked in the top 4, the improvements are still less significant. Indeed, whereas with the simple docking, the number of targets with correct top-4 rank poses was in the interval [57, 75] (Table 1, column 4), after rescoring, this number was in the interval [62, 76], the highest value being obtained with the sampling and rescoring with Glide. Surprisingly, whereas the poses obtained with Surflex were the closest to the X-ray positions, rescoring them with other scoring functions did not significantly help in top-ranking them. Indeed, Surflex was able to correctly position the ligand for 84 targets (Table 1, column 3), and the best top-4 ranking of these poses was obtained with *Goldscore* and *FlexX*, for 75 targets, which is only slightly better than the initial docking results obtained by *Surflex* scoring, with 72 targets (Table 1, column 4). For 3 of the targets (hivpr, pde5a and xiap), whose ligands were correctly positioned with Surflex, none of the rescoring programs was able to rank the correct poses between the top 4, so what about the X-ray poses themselves? Would they be well ranked?

To answer this question, the crystal structure poses were rescored following the same procedure as above, with a slight optimization to remove clashes, and ranked among the other poses. The results are given in Table 4, where we can observe the poor ranking given by the programs.

**Table 4:** Assessment of the rescoring results for the X-ray poses

	Rescoring			
	*Glidescore*	*Surflex*	*Goldscore*	*FlexX*
X-ray in top 1	6	6	9	6
X-ray in top 4	23	13	42	27

Number of targets whose X-ray pose is ranked in top 1 or top 4, when added to the pool of poses for rescoring

Less than 10 crystal ligands were top-ranked and only between 13 (with *Surflex*) and 42 ligands (with *Goldscore*) were ranked in the top-4 poses. However, the X-ray pose is ranked above all incorrect ligand poses (whose RMSD ≥ 2 Å) for 55 targets by Glide and Gold, for 33 targets by FlexX and for 30 targets by Surflex. This average result could be due to the quality of the scoring functions, but also to the quality of the crystal structures for which the slight optimization may not be sufficient to satisfy the scoring functions energy criteria. Note that for 2 (fnta and fkb1a) of the 6 hard targets cited above, for which the best poses were incorrect, the ligand crystal structure was ranked between the top-4 poses by *Glidescore*, showing that for these 2 targets, the failure of docking is not due to the scoring function but to the conformational sampling.

Despite these observations, the ranking of the ligands that were positioned by the programs and which therefore satisfy the scoring functions energy criteria still has to be improved. For this purpose, for each target, all the poses obtained with the four programs (up to 120 poses/target) were gathered in one pool and re-ranked. The results are given in Table 3, last line. In this pool, there are 94 targets with a correct best pose given by at least one of the four programs. Therefore, it is not surprising that the number of targets with a correct top-rank pose is significantly improved compared to docking, ranging from 63 to 75 targets, to be compared to the interval [52, 65] for docking. In addition, in this case, the best poses are not ranked far, since in the top-4 poses there is at least one correct pose for a number of targets in the interval [72, 86], which is significantly higher than that obtained with docking, [57, 75]. This means that by limiting the analysis of the results to the top-4 ranked poses, one may have between 57 and 75% chance of finding the right ligand position by docking and between 72 and 86% chance by rescoring the pool poses, the latter being obtained by *Goldscore*. In other words, to obtain an honorable yield, one needs to use several docking programs and rescore all the poses gathered in one pool, which represents a heavy, time-consuming procedure. A much easier and rapid one may be used, the United Subset Consensus (USC).

#### USC method

The USC method is based on the observation that there are no correlations between the RMSDs obtained with the different programs, neither considering their best poses nor their top-rank ones. This observation was presented above, in the section “Evaluation of the docking programs” (Figs. 3, 4). USC applied to docking consists of gathering, for each target, a subset of 4 poses made of the top-rank ones taken from each of the 4 programs, then the 4 second-rank poses, etc. Therefore, contrarily to docking or rescoring, with USC the poses are considered four by four. Because of the absence of correlation between the results of the programs, and therefore their variety, using USC by considering a subset made of the union of the four programs results may improve the yield, as it was observed in [34]. Here, for the sake of comparability with the efficiency of the programs, for each target, only the first USC element, made of the 4 top-rank poses, is compared to the top-4 poses ranked by the programs. USC was applied to both the results of docking and rescoring. Whereas USC based on the rescoring did not improve the results as observed in Table 3B, USC based on the results of the initial docking brings a spectacular improvement. Indeed, in this case, 87 targets were found to have at least one pose with RMSD < 2 Å from the X-ray structure (Table 1, last line), which has to be compared to the docking range [57; 75]. This result is better than any of the ones obtained for the top-4 poses whether with docking or rescoring, [57; 86], although it is close to the rescoring results when considering the pool of poses rescored by either *Goldscore* or *Glidescore*. However, the advantage of USC is its rapidity and ease of use compared to the rescoring procedure, which is long and fastidious, and it increases the chance of finding the good pose, within only 4 poses, by 12–30% compared to docking.

### Are all the top-ranking failures due to scoring functions?

To answer this question, let us consider again the pool of poses used in the rescoring subsection. As presented above, there are 94 targets in this pool with at least one correct best pose, obtained by one program or another. Of these targets, 9 could not be top-ranked by any of the four programs. They are colored in pink, in Additional file 2: Table S1. A close examination of these targets shows various reasons for the ranking failure. The observations are summarized in Table 5, where are reported the RMSD of the best best-pose obtained by any of the four programs, and the RMSD of the best top-score pose, which consists of the top-score pose with the smallest RMSD, among the four top-score poses.

**Table 5:** Analysis of the reasons for the 9 targets ranking failure

Target	RMSD (Å) of the best best-pose	RMSD (Å) of the best top-rank pose	Reasons for incorrect ranking	Due to the scoring functions?	Possible recommendations
cp3a4	2.03 Surflex	4.14 *Glidescore*	Coordination bond	No	
xiap	1.25 Surflex	3.78 *FlexX*	Ligand not well anchored in the binding site	No	
hdac8	0.91 Glidescore	2.91 *Goldscore*	The metal ion chelation is not considered explicitly or not well parametrized	Yes	With modified parameters of the metal, the top-rank pose is correct
hs90a	0.32 Gold	9.54 *Glidescore*, *FlexX*, *Goldscore*	An off-center pose is privileged	No	Possibility of the existence of a secondary site difficult to reach (dynamical considerations)
igf1r	0.72 Surflex	2.32 *Surflex*	Difference in protonation	No	Include in situ protonation in the docking procedure
cxcr4	1.94 Gold	2.95 *Surflex*	Difference in protonation	No	Similar to igf1r
pnph	0.65 Gold	3.14 *Glidescore*	Difference in protonation	No	Similar to igf1r
hivpr	1.97 Surflex	2.26 *Glidescore*, *Goldscore*	The difference is located in a loose or exposed to solvent part of the ligand	No	The difference is not a problem
tysy	1.27 Surflex	2.7 *Goldscore*	The difference is located in a loose or exposed to solvent part of the ligand	No	Similar to hivpr

For each ligand, all poses of the pool were ranked by each program and we only consider the top-rank pose with the smallest RMSD, without any consideration of the program

1. Two targets seem not to be well adapted for docking: **cp3a4**, whose best pose was considered as correct because it is very close to the limit RMSD of 2 Å. It is a cytochrome and its ligand establishes a coordination bond with the iron atom of its heme. Such an interaction is not well handled by docking programs, for which the right pose presents a high van der Waals repulsion. **xiap** has a ligand that is not anchored in the binding pocket, but binds superficially between two adjacent proteins in the crystal lattice (Fig. 7a). Therefore, considering the small number of protein–ligand interactions, it is not surprising that the best pose was not top-ranked.

**Fig. 7:**
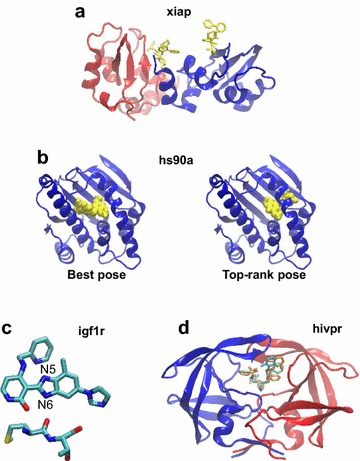
Representative structures to illustrate the top-ranking failure which is not due to scoring functions. **a** Structure of the crystallographic dimer of xiap, where chain A is in *blue* and chain B in *red*. The ligand is in *yellow sticks*. It is obvious that there are too few contacts between the protein and the ligand to allow the latter anchorage. **b** Structure of hs90a in *blue* with the ligand in *yellow spheres*. *Left* the ligand is in its best pose (RMSD = 0.32 Å), in the crystallographic binding pocket. *Right* the ligand is in the best off-center pose (RMSD = 9.54 Å), top-ranked by Glide, Gold and FlexX after the rescoring of the pool of poses. **c** Crystal structure of the ligand of igf1r with its closest protein environment. It shows the necessity of protonating the N6 atom to allow it to establish a hydrogen bond with a carbonyl from the protein backbone. The carbon atoms are in *cyan*, the nitrogen in *blue*, the oxygen in *red* and the sulfur in *yellow*. **d** hivpr, with chain A in *blue* and chain B in *red*. The crystal ligand is in the same color code as (**c**) and the top-rank pose is in *orange*. Their comparison shows that the discrepancies are located in the accessible to solvent part of the ligand, which has no consequence on the results. VMD software [45] was used for molecular visualization
  For three targets, hdac8, hs90a and igf1r, the correct top-rank pose was found after docking but not after rescoring. For hdac8, it was found by Gold when scoring with *PLP* and for hs90a and igf1r, by Glide with *Glidescore*. There is a variety of reasons why the rescoring did not top-rank a correct pose.
2. For **hdac8**, the ligand participates in the chelation of a zinc ion in its proximity. In the scoring function *PLP*, there is a specific term for the metal, which is not the case in *Goldscore*. This may explain why the top-rank pose was correct after docking but not after rescoring. In addition, in *PLP* the metal term parameters [35] are different from those of *ChemScore* [31] and the other functions (*Glidescore* and *FlexX*) based on it. When rescoring the poses of the pool with *PLP*, the best pose was top-ranked by this scoring function. Incidentally, hdac8 was the only target for which the result of rescoring was better with *PLP* than with *Goldscore*.
3. For **hs90a**, in docking, only Glide succeeded in top-ranking a correct pose (RMSD = 0.46 Å). But this program was confused during the rescoring process by the presence of poses coming from FlexX, relegating its top-pose to lower ranks. Indeed, FlexX created poses at the edge of the binding site. When these poses were added to the pool, an off-center pose was privileged by Glide, Gold and FlexX itself, with an RMSD of 9.54 Å (Fig. 7b). Since visually it was not obvious why the crystal position would be better than this off-center pose, we minimized the energy of all protein–ligand complexes, using the CHARMM36 force field [36] for the protein and the CHARMM General Force Field (CGen) [37] for the small molecule. These minimizations confirmed the results of the scoring functions, i.e., most of the off-center pose complexes had the lowest energies and the best pose (RMSD = 0.32 Å) was in the 59th rank. In addition, the first correct pose (RMSD = 1.88 Å) was in the 15th rank. This is due to the fact that there are more interactions between the protein and the ligand in the off-center pose than in the crystal pose. Therefore, in such a case, modifying the scoring functions does not seem to be the solution for improving the detection of the right pose. The difference between these poses could be due to entropy considerations or to dehydration of the binding site during the ligand binding, but this does not seem likely considering the conformations of the ligand and the sites. However, it appears more likely that the ligand could encounter dynamical difficulties while reaching the off-center pocket, which can be monitored by directed molecular dynamics, out of the scope of the present article. Finally, there is also a small probability that both poses are possible and the crystallization conditions have privileged one of them.
4. Some artifacts are observed in the upstream preparation of the ligands. For **igf1r**, there is a problem in the protonation of the ligand N5 and N6 nitrogen atoms (Fig. 7c). It is obvious from the crystal structure that the proton should be on N6 to allow the establishment of a hydrogen bond with the protein. However, the ligand provided on the DUD-E website [27] is protonated on N5. The ligand preparation programs, like MarvinSketch (Marvin 6.1.6, 2014, http://www.chemaxon.com) or LigPrep (Schrödinger Release 2016-4: LigPrep, Schrödinger, LLC, New York, NY, 2016), show that N5 and N6 have the same pKa, and depending on the prior assignment of the bonding order, the protonation is made on either one or the other nitrogen. A posteriori, we have monitored the protonation of this ligand using these two programs starting from either the SMILES format or the pdb coordinates. In all cases, by default, the protonation is made on N5, and the protonated N6 was not proposed among all possible tautomers of this ligand at pH 7 ± 2. To obtain a protonation on N6, the bond order should be given explicitly and manually in this sense. Then, the protonated N6 is obtained as the most populated species and N5 is not present among the proposed tautomers, showing an issue in the protonation procedure of the programs. Therefore, in the case of no prior knowledge of the ligand pose, as simulated here, only the N5 protonated ligand is given for docking and it is not surprising that the scoring functions favor a pose where the ligand is slightly shifted in order to avoid repulsive electrostatic forces. To avoid such an artifact, it would be preferable that the protonation of a ligand is made during the docking procedure, in order to adapt it by considering the protein environment. A similar problem was observed for **cxcr4** and **pnph**.
5. In some cases, the relatively high value of the RMSD of the top-score pose does not present any drawback because the difference with the correct pose is only located in a loose or solvent-accessible part of the ligand. This is the case of **hivpr** (Fig. 7d) and **tysy**.

These observations show that for only one of the nine targets, hdac8, the problem of top-ranking incorrect poses could be resolved by improving the scoring functions.

## Conclusions

In this study, we present the results of the evaluation of 4 docking programs, Glide, Gold, Surflex and FlexX. All the conclusions are only applicable to semi-rigid docking, where the protein structure is fixed in the favorable conformation, since it is taken from the crystal structure of the complex and the ligand starting structure is the energy minimum conformer. We observe that, generally, the programs present good performances in positioning correctly the ligand in the binding site. This is especially true for Surflex, with its 84 well-positioned ligands. Altogether, the programs positioned correctly the ligands of 94 targets over 100. However, they have some difficulties in the ranking of the generated poses, indicating some weaknesses in the scoring functions. Apart from the number of rotatable bonds of the ligands, which is related to their molecular weight, there is no correlation between either the scoring functions or the sampling procedure of these docking programs and the main physicochemical properties of the ligands or the proteins. Consequently, there is no particular program or protocol that may be recommended for specific proteins or ligands, although some programs seem to work globally better than the others, like Glide and Gold, with a correct top-rank ligand for more than 60% of the targets. This performance is much better than that observed in virtual screening with the same programs, where a good BEDROC value (over 0.5) was obtained for less than 30% of the targets. In fact, for each program, the comparison of its performance for docking and virtual screening showed the absence of correlation between these two aspects of the work. A well-positioned and top-ranked ligand in docking does not guarantee its good ranking during VS, and vice versa. Therefore, improving the scoring functions for docking may be necessary but clearly insufficient for improving the virtual screening performance.

Similarly to our recommendations for VS [26], we recommend for docking the use of several programs and the combination of their results. This is another validation of the idea of “the wisdom of crowds” known in social sciences [38], which suggests that when there are too many good possibilities from different sources, collect the wisdom of all of them to reach a global solution (this remark was given by one of the article reviewers). Here, this collection was done either by gathering all the poses in a pool and rescoring them with the programs or, still better, by the use of USC based on the docking results. With rescoring, the best results were obtained by *Glidescore*, which yielded a correct top-rank pose for 75 complexes, and *Goldscore*, which gave at least one correct pose in the top-4 rank poses for 86 complexes. USC allowed us to obtain a similar number of correct top-4 rank poses, for 87 complexes, but in a much easier and faster way. Despite the apparent weakness of each individual scoring function, the performance of the four docking programs was surprisingly remarkable when taken altogether. Indeed, when all poses of the 100 complexes are put in one pool, for 86 of them at least one program could top-rank a correct pose, and for 9 other complexes, at least one correct pose was found by the search algorithms but all four scoring functions failed to top-rank them and finally, for the remaining 6 complexes, no correct pose was found during the sampling process, although the crystal structure was in the top-4 rank for 2 of them. From the close observation of the 9 complexes with successful conformational sampling and failed ranking, it is clear that the failure is not necessarily due to the scoring functions, but also to other considerations, like the unsuitability of the complex for docking, the neglect of dynamical approaches of the ligand to the protein pocket, the protonation of ligands apart from the protein environment and finally, all poses with relatively high RMSD are not necessarily incorrect when the divergence only concerns the out-of-pocket part of the ligand. Therefore, to improve the results of docking, we must consider the problem from different angles, the most innovative being the dynamics of the ligand to reach the pocket and the protonation of the ligand (and maybe the protein) in its environment.

## Methods

### Preparation of the DUD-E database

#### Preparation of the DUD-E targets and definition of the binding sites

The crystal structure of the proteins were taken from the DUD-E dataset (http://dude.docking.org ) and prepared using the Protein Preparation Wizard from the Schrödinger suite (http://www.schrodinger.com) as previously described [26]. The obtained mol2 files, with the correct atom types, were used for the four docking programs. To define the binding site, all residues of the protein structure with at least one heavy atom within 5 Å from the ligand were selected. Then, this selection was refined upon individual visual inspection, by adding, when necessary, residues beyond 5 Å that are essential for the continuity of the cavity.

#### Preparation of the crystal ligands

The mol2 files of the co-crystallized ligand structures were taken from the DUD-E dataset, in which, for each ligand, the major protonation state was given at pH 7. However, for few ligands, the structures had to be curated to ensure the attribution of the correct bond orders and atom types. This was done on the basis of the information given by the articles that accompany the X-ray structures. In order to randomize the 3D structures of the ligands, all the mol2 files were converted to the 2D SMILES format and the Ligprep module of the Schrödinger suite was used to generate a starting 3D optimized conformation for each ligand, using the force field OPLS2005.

### Docking and rescoring methods

In order to obtain comparable results with all four programs, we used the docking method that they have in common, the semi-rigid docking, in which the target atoms are fixed and the ligands are flexible. In order to avoid favoring any of the programs, the default parameters were used for the docking setups. The same procedure was followed for all targets: each program was requested to generate thirty distinct poses, separated by an RMSD greater than 0.5 Å.

#### Glide

Glide [4, 29] version 6.8 (Schrödinger) was used. Glide docking requires the generation of a cuboid grid centered on the binding site. For this purpose, the rotation of the target was done when necessary and the grid dimensions were adjusted visually to fit the cavity shape. Both the standard precision (SP) [4] and the extra precision (XP) [39] docking modes were used with the *Glidescore* and *E*-*model* scoring functions [4].

The rescoring of the poses was performed using the “mininplace” docking option, which ensures that the poses are refined locally (minimized in place).

#### Surflex

The docking with Surflex 2.745 [5, 30] was performed using the “GeomX” mode, which increases the docking accuracy. The docking procedure necessitates the generation of a “protomol”, which consists of a set of hydrophobic and hydrophilic probes (CH4, NH and CO) that completely fit the cavity surface, making all possible interactions with the binding site residues. The generation of the protomol was based on the binding site residue list previously defined.

The rescoring of the poses was performed using the “score_list” command. This procedure automatically includes a local optimization of the poses.

#### Gold

Gold [3, 32] version 5.2.2 from the Cambridge Crystallographic Data Center (CCDC) was employed. The binding site residues were explicitly specified, as well as the metal ions coordination geometry. The latter was obtained by the prediction module of Gold, combined to the bibliographic information about the ion and its surrounding amino acids. The conformational sampling is based on a genetic algorithm and the scoring functions were *PLP* [35, 40] and *Goldscore* [3, 32].

The rescoring of the poses was performed using the standard procedure with the simplex option, where the poses were optimized and rescored. Both *PLP* and *Goldscore* scoring functions were considered.

#### FlexX

FlexX [6] version 2.1.5 (BiosolveIT) was used. The binding site residues were explicitly specified, as well as the metal ions coordination geometry, similarly to what was done for Gold. In FlexX, the small molecule search algorithm is fragment based. The selection of the base fragments was set to automatic mode by using the “selbas a” option, where “a” stands for “automatic”, and the placement of the fragments used the standard algorithm (option 3). Each resulting pose was optimized by up to 1000 steps of energy minimizations, with an additional cutoff distance of 3 Å, to allow more interaction partners.

The rescoring of the poses was performed after a local optimization of the pose with 1000 iterations of the local optimizer.

### RMSD calculation

The RMSD measures the difference in conformation and position between two poses of a molecule. However, for symmetrical molecules, the symmetric atoms do not have the same name, which generates an artificially high RMSD. Therefore, a symmetry corrected RMSD was calculated using rmsd.py, a python script available in the Schrödinger suite, which allows us to overcome the symmetry problem. Only the heavy atoms were considered.

### Target characteristics

#### Target families

Only fifty-eight of the 102 targets could be clustered in nine families. The others were kept as miscellaneous proteins. To define the protein families, their sequences and 3D structures were compared. All pairs of proteins with more than 20% sequence identity, with homologous 3D structures and with similar functions were gathered into families. More details are given in [26].

#### The binding site properties

Three properties of the binding site were considered: the total number of atoms at 4 Å from its surface, its hydrophobicity, i.e., the fraction of carbon atoms among this total number, and its exposure to the solvent. In order to define the surface of the binding site, the protomol generated by Surflex (see above), which closely covers the site, was used. All the heavy atoms of the protein, situated at less than 4 Å from the protomol, were considered as the surface of the cavity. The exposure of the binding site was calculated by using the SiteMap package [41] version 3.3 (Schrödinger). It measures the degree of openness of the site to the solvent. For more details, see [26].

### Crystal ligand properties

The physicochemical properties of the ligands were calculated using the canvasMolDescriptors [42] module from the Schrödinger suite. They were all taken directly from the results, except for EC (embranchment count), which was calculated by summing the number of atoms involved in three or more covalent bonds. The procedure is detailed in [26].

### Statistics

The Spearman correlation coefficients, the Shapiro–Wilk normality test, the parametric Student *t*-test and the non-parametric Mann–Whitney–Wilcoxon test were calculated using the R program [43].

### Energy minimizations

All 120 protein–ligand structures of hs90a were energy minimized using CHARMM program [44], with the CHARMM36 force field [36] for the protein and the CHARMM General Force Field (CGen) [37] for the small molecule. This minimization was done with 5000 steps of steepest descent, followed by other 5000 steps of conjugate gradient algorithms. The electrostatics and van der Waals energy terms were truncated using the switching function with a cutoff distance between 6 and 10 Å, and the dielectric constant was equal to 2 times the distance between the interacting atoms.

### Figures

Visual Molecular Dynamics (VMD) [45] was used for the protein images, R [43] for Figure 5 and Kaleidagraph version 4.5.0 (http://www.synergy.com/) for all the other plots.

## Additional files

**Additional file 1:** Four supplementary figures. They show the distribution of the protein (**Figure S1**) and small molecule (**Figure S2**) properties, the comparison of two scoring functions of Gold and Glide for docking and rescoring (**Figure S3**) and finally, the comparison of the properties for easy and hard targets (**Figure S4**).

**Additional file 2: Table S1.** Table gathering the results of docking and protein and ligand properties. The target name from DUD-E is given in column A. The difficulty of docking and ranking, column B, is colored in green for the 28 easy targets (top-ranked with all programs), in red for the 6 hard targets (with no correct best pose found with any program), in pink for the 9 best but not top-rank targets exposed in Table 5 (a correct best pose found by at least one program but not top-ranked with any scoring function when rescored in one pool). The rest is in gray. The protein properties are given in columns C to G and the ligand properties in columns H to O. Then follow the RMSDs of the top-rank and best poses obtained when docking with Glide (columns P, Q), Surflex (R, S), FlexX (T, U) and Gold (V, W).

## Abbreviations

DUD-E: Database of Useful Decoys-Enhanced
RMSD: root-mean-square deviation
FCA: fraction of carbon atoms
MW: molecular weight
PSA: polar surface area
EC: embranchment count
HBA: hydrogen bond acceptors
HBD: hydrogen bond donors
RB: rotatable bonds
RC: ring count
PLP: piecewise linear potential
VS: virtual screening
BEDROC: Boltzmann enhanced discrimination of receiver operating characteristic
USC: United Subset Consensus
