# Supplementary material for: Efficient conformational sampling and weak scoring in docking programs? Strategy of the wisdom of crowds
Source: J Cheminform. 2017 Jun 12;9:37. doi: 10.1186/s13321-017-0227-x (PMC5468358; doi:10.1186/s13321-017-0227-x)
Supplement: Supplementary file 1 — Additional file 1: Four supplementary figures. They show the distribution of the protein (Figure S1) and small molecule (Figure S2) properties, the comparison of two scoring functions of Gold and Glide for docking and rescoring (Figure S3) and finally, the comparison of the properties for easy and hard targets (Figure S4). [file 13321_2017_227_MOESM1_ESM.pdf]

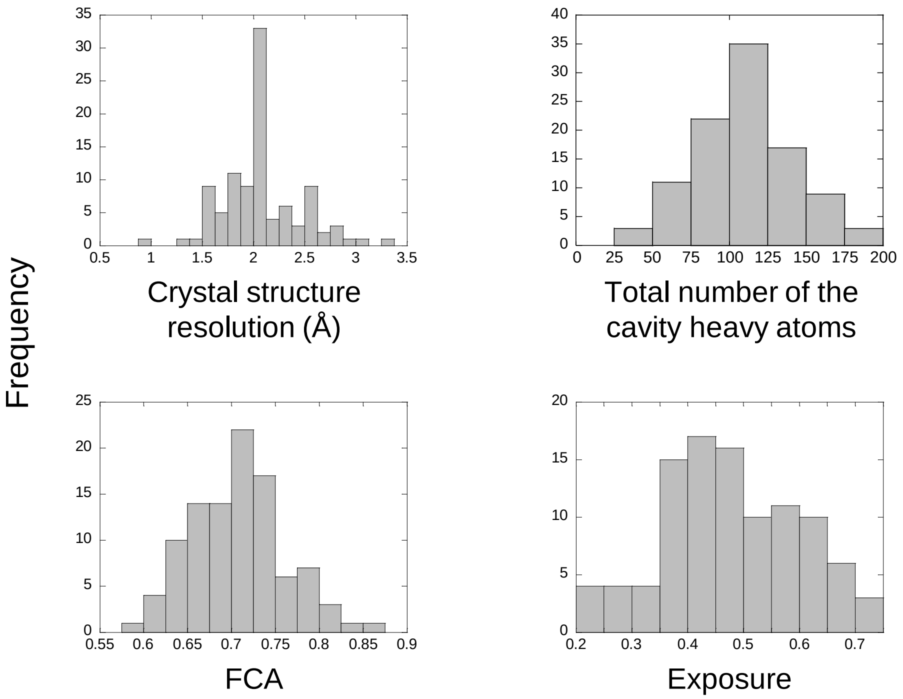

Figure S1. Distribution of the protein properties. The crystal structure resolution represents the accuracy of the structure. The total number of the cavity heavy atoms represents the size of the cavity. FCA is the fraction of carbon atoms among the heavy atoms of the cavity surface, which corresponds to its degree of hydrophobicity. And finally, the exposure gives the degree of openness of the cavity.

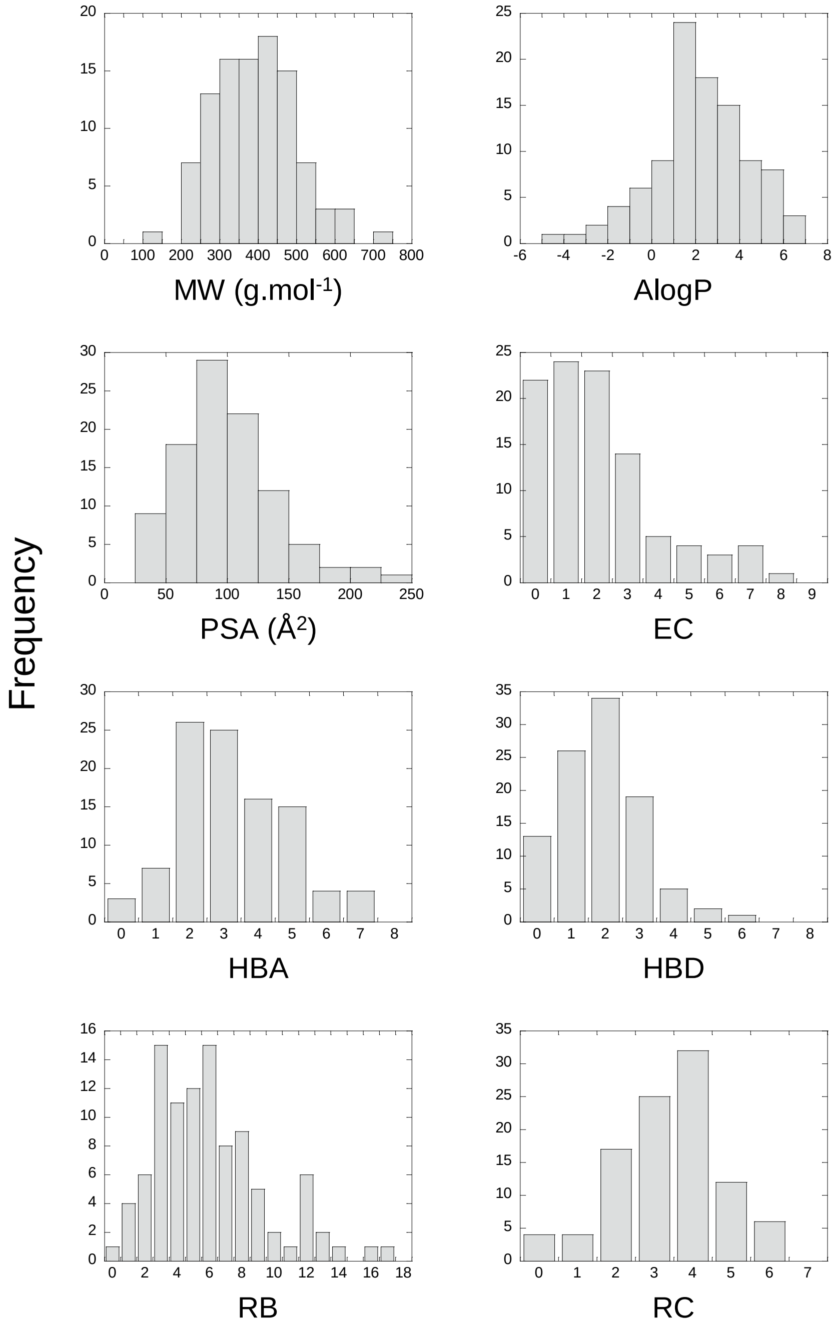

Figure S2. Distribution of the small moecule properties. The molecular weight (MW), the octanol/water partition coefficient (AlogP), the polar surface area (PSA), the embranchment count (EC), the number of hydrogen bond acceptors (HBA), the number of hydrogen bond donors (HBD), the ring count (RC) and the number of rotatable bonds (RB)

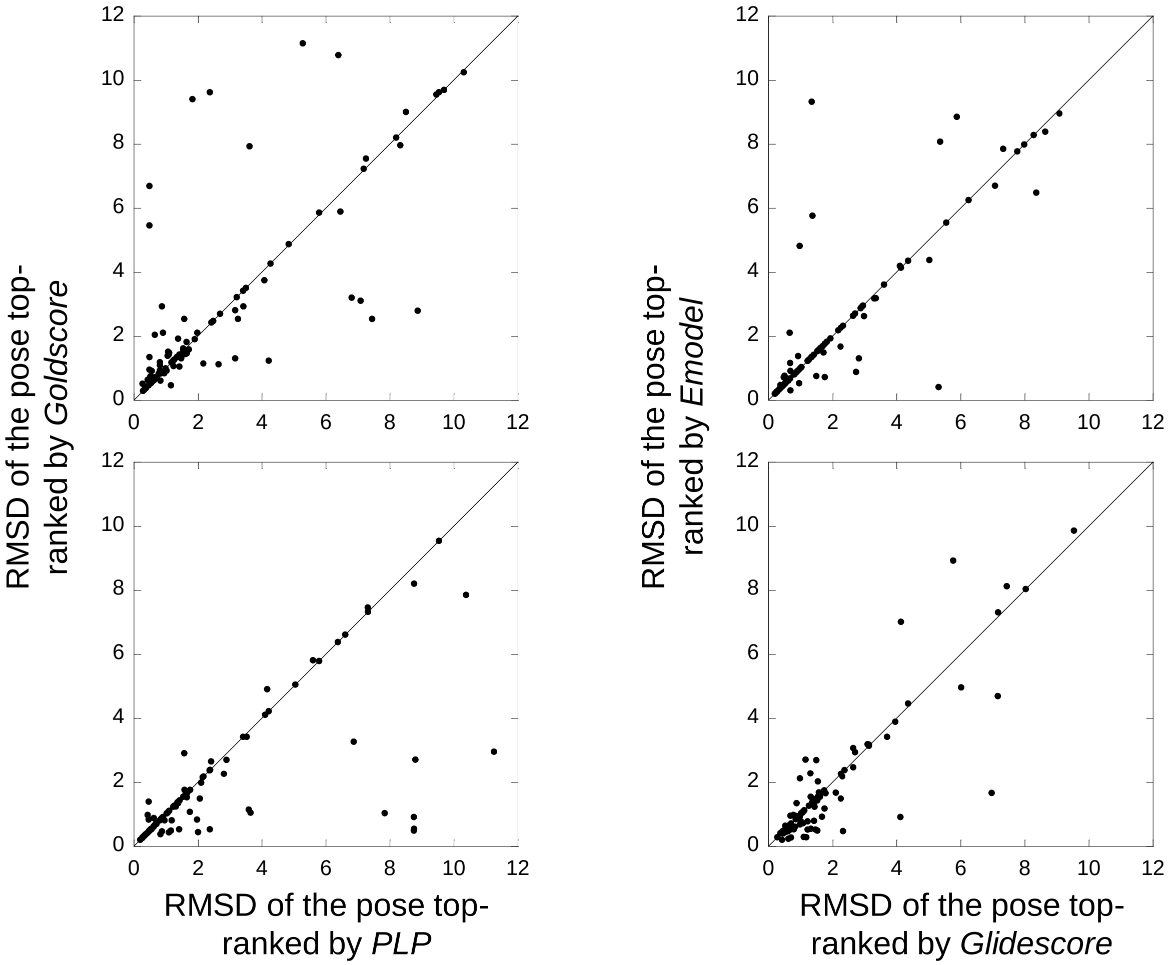

Figure S3. Comparison of two scoring functions of Gold and of Glide. For each target, the RMSD (Å) of the top-rank pose found by one scoring function is given with respect to the RMSD of the top-rank pose found by the other scoring function of the same program. Results of docking, *upper panels*, and rescoring of the poses obtained by the same program, *lower panels*. When the points are more dispersed under the diagonal, the scoring function corresponding to the X-axis presents a weaker performance as to top-ranking a correct pose than the scoring function of the Y-axis, and *vice versa*. It can be observed that for docking, *PLP* is better than *Goldscore* and *Glidescore* than *Emodel*, whereas for rescoring, *Goldscore* is better than *PLP* and *Glidescore* and *Emodel* are similar.

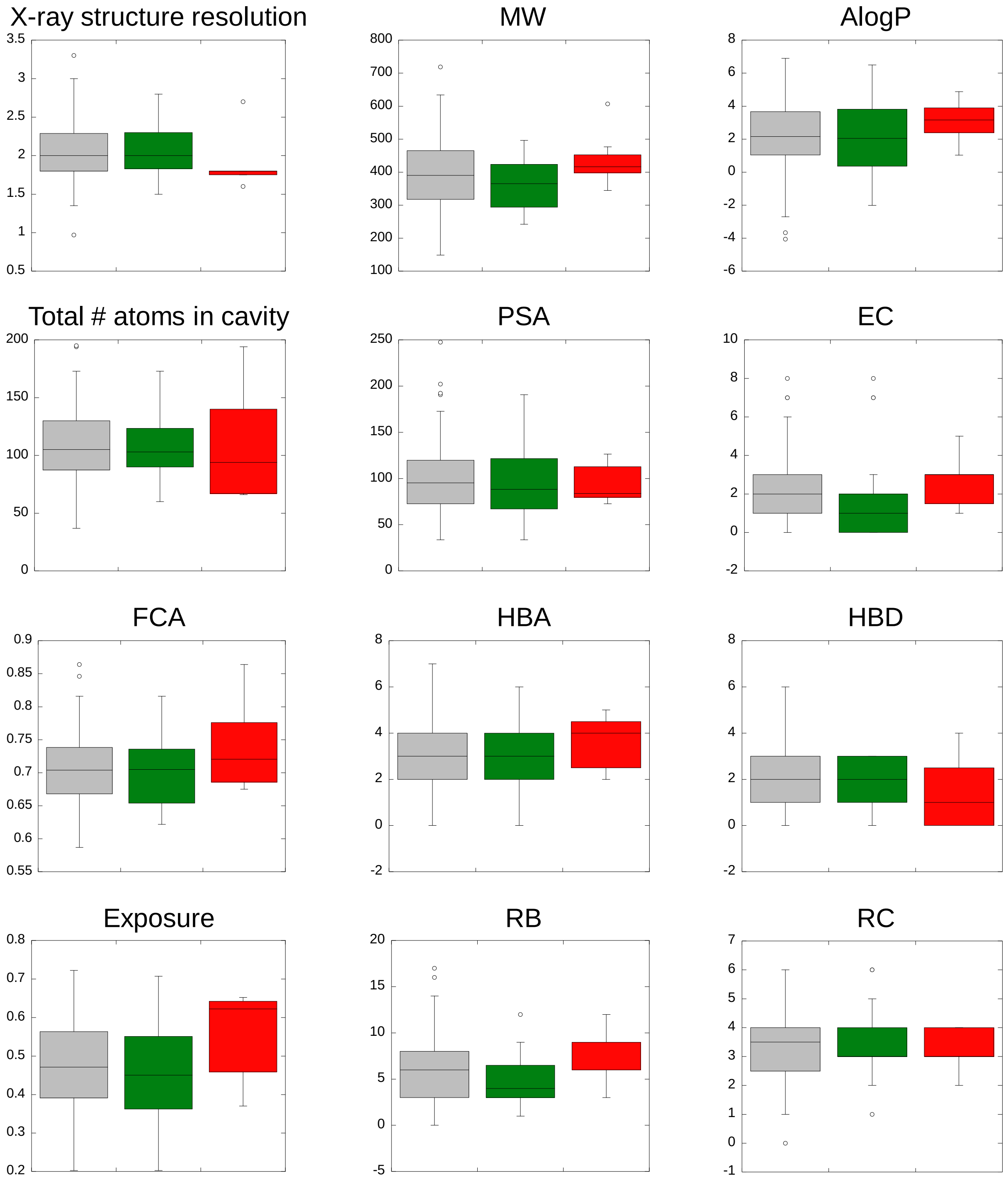

Figure S4. Comparison of the properties for easy and hard targets. The Box plots for each protein (left panels) and ligand (middle and right panels) property are in gray for all targets (100 targets), in green for easy targets (28) and in red for hard targets (6). Each box encloses 50% of the data with the median value of the variable displayed as a line. The separated points exceed (positively or negatively) the box value by 1.5*Inter Quartile Distance. The Shapiro-Wilk test was done to assess the normality of each property distribution. For normal distributions, the Student's *t*-test was used, otherwise, the Mann-Whitney-Wilcoxon test was used to compare the difference between easy and hard targets. No significant difference was found, based on a significance threshold of 1%.
